# Impact of the Regional Network for AMI in the Management of STEMI on Care Processes, Outcomes and Health Inequities in the Veneto Region, Italy

**DOI:** 10.3390/ijerph15091980

**Published:** 2018-09-11

**Authors:** Mario Saia, Domenico Mantoan, Marco Fonzo, Chiara Bertoncello, Marta Soattin, Milena Sperotto, Tatjana Baldovin, Patrizia Furlan, Maria Luisa Scapellato, Guido Viel, Vincenzo Baldo, Silvia Cocchio, Alessandra Buja

**Affiliations:** 1 Medical Directorate, ULSS 6 Euganea, Veneto Region, Via Enrico degli Scrovegni 14, 35131 Padua, Italy; mario.saia@sanita.padova.it; 2 Health and Social Services, Veneto Region, Dorsoduro, 3493—Rio Nuovo, 30123 Venice, Italy; domenico.mantoan@regione.veneto.it; 3 School of Specialization in Hygiene, Preventive Medicine and Public Health, University of Padua, Via Loredan 18, 35128 Padua, Italy; marco.fonzo@studenti.unipd.it (M.F.); marta.soattin@studenti.unipd.it (M.S.); 4 Department of Cardiological, Thoracic and Vascular Sciences, Hygiene and Public Health Unit, University of Padua, Via Loredan 18, 35128 Padua, Italy; milenasperotto1978@gmail.com (M.S.); tatjana.baldovin@unipd.it (T.B.); patrizia.furlan.73@gmail.com (P.F.); vincenzo.baldo@unipd.it (V.B.); silvia.cocchio@unipd.it (S.C.); alessandra.buja@unipd.it (A.B.); 5 Department of Cardiological, Thoracic and Vascular Sciences, Occupational Medicine Unit, University of Padua, Via Loredan 18, 35128 Padua, Italy; marialuisa.scapellato@unipd.it; 6 Department of Cardiological, Thoracic and Vascular Sciences, Legal Medicine Unit, University of Padua, Via Loredan 18, 35128 Padua, Italy; guido.viel@unipd.it

**Keywords:** health care research, quality assurance, hospital management, health inequities

## Abstract

Cardiovascular diseases are a leading cause of death in Europe. Outcomes in terms of mortality and health equity in the management of patients with ST-Elevation Myocardial Infarction (STEMI) are influenced by health care service organization. The main aim of the present study was to examine the impact of the new organizational model of the Veneto Region’s network for Acute Myocardial Infarction (AMI) to facilitate primary percutaneous coronary intervention (PCI) on STEMI, and its efficacy in reducing health inequities. A retrospective cohort study was conducted on HDRs in the Veneto Region for the period 2007–2016, analyzing 65,261 hospitalizations for AMI. The proportion of patients with STEMI treated with PCI within 24 h increased significantly for men and women, and was statistically much higher for patients over 75 years of age (APC, 75–84: 9.8; >85: 12.5) than for younger patients (APC, <45: 3.3; 45–64: 4.9), with no difference relating to citizenship. The reduction in in-hospital, STEMI-related mortality was only statistically significant for patients aged 75–84 (APC: −3.0 [−4.5;−1.6]), and for Italians (APC: −1.9 [−3.2;−0.6]). Multivariate analyses confirmed a reduction in the disparities between socio-demographic categories. Although the new network improved the care process and reduced health care disparities in all subgroups, these efforts did not result in the expected survival benefit in all patient subgroups.

## 1. Introduction

Cardiovascular diseases are a primary cause of death in Europe, responsible for more than half of all deaths across the region, with heart disease or stroke the leading cause of death in all 52 countries [1]. Ischemic heart disease now accounts for almost 1.8 million deaths a year, or 20% of all deaths in Europe, albeit with large variations between countries [2]. There has been an overall declining trend in the mortality due to ischemic heart disease over the past three decades, however [3].

Outcomes in patients with ST-Elevation Myocardial Infarction (STEMI) are influenced by many factors, some of which could relate to how health care services are organized, such as the availability of emergency medical services based on STEMI networks, which can have an impact on the delay before a patient is treated. In patients clinically suspected of having myocardial ischemia and ST-segment elevation, reperfusion therapy needs to be initiated as soon as possible [4,5]. In the literature, some studies have shown that obtaining an electrocardiogram (ECG) before reaching the hospital significantly reduces the door-to-balloon time, and that subsequently admitting ambulances directly to the cardiac catheterization laboratory (CCL), bypassing the emergency room (ER), substantially reduces the time to primary percutaneous coronary intervention (PCI) [6,7], resulting in a significant time saving for patients [4,5]. Changes in health care service organization such as prehospital activation have led to the patients involved having the highest ejection fractions, and the shortest hospital stays [8], and other studies have reported a reduction in mortality too [9].

According to the literature, myocardial infarction management may differ in relation to patients’ characteristics, such as age, race and gender [10,11,12], and whether disparities in access to cardiac procedures translates into a different mortality risk is not known [13]. In recent years, there have been national and global efforts to rectify such health inequities, but few studies have investigated how successful they have been. One framework for action defined six strategies imperative for eliminating disparities in cardiovascular health care [14], and one of these strategies involves collecting health care data by ethnicity and gender to orient program development, implementation and assessment [15].

A study conducted at one American clinic showed that a systems-based approach to STEMI care reduced gender disparities and improved STEMI care and outcomes in women [16]. In another larger American study it emerged that a state-wide STEMI regionalization program was associated with improvements in treatment times for female, black, and elderly patients comparable with those for middle-aged, white, male patients [17]. Finally, in a recent European population-based study, socioeconomic inequity of access to revascularization was no longer apparent following the redesign of revascularization services in the South Wales Cardiac Network [18]. Such studies have been rather scarce in the European context, however. Hence the present study to assess the impact of the network implemented for AMI in the Veneto Region over the past 10 years (based on a ‘hub and spoke’ organizational model) in terms of the quality and outcome of the care process, and its efficacy in reducing health inequities. 

## 2. Materials and Methods

### 2.1. Geographical and Socio-Demographic Context

The Veneto Region is located in the north-eastern part of Italy and is the eighth largest region in Italy with a total area of 18,398.9 km^2^. In terms of population, on 1 January 2017, the Veneto Region was the fifth largest Italian region, with just over 4.9 million inhabitants and approximately 485,000 immigrants residing in the region, accounting for 9.9% of the total population [19]. In common with the rest of Italy, the main demographic feature of the Veneto Region is its aging population: some 20% of the Veneto population is over 65 years old and 10% are over 75. It is one of the most affluent regions in terms of per-capita income, with only 6% of families living in relative poverty [20]. A quarter of the population has a chronic health problem, including 65% of those over 65, and more than 25% of those aged 75–84; and more than 57% of the population over 75 are disabled [21].

### 2.2. Health Care System

The Italian health care system is essentially region-based [22]. In the Veneto Region, the regional authorities take full responsibility for organizing and administering public-financed health care through their regional health departments in accordance with a national health plan designed to assure an equitable provision of comprehensive care throughout the country. Responsibility for planning health care is shared by the central government and the regional authorities. 

The regional authorities coordinate and control local health units (LHU) (Regional Law No. 19/2016), each of which is a separate unit within the National Health System (NHS) that plans and delivers health care services to its local community, based on a regional health plan, and determines the regional health care reorganization, specific for each region.

In the Veneto Region, the hospital network includes seven large “hubs” with regional or provincial catchment areas, and 20 local “spokes” each serving around 200,000 residents. There are also 40 smaller “node-in-the-net” hospitals that provide integrative or specific health services (such as mental health services or rehab clinics).

The LHUs are responsible for assessing needs and providing comprehensive care to their local population, either using their own staff and facilities or contracting the services out to public hospital enterprises and for-profit and non-profit independent hospitals and specialist outpatient service providers. Private providers must be accredited and have a contract with the LHU.

Tax contributions allocated to the National Health Fund are redistributed horizontally between the regions using a weighted capitation mechanism. LHU services are financed by the regional governments. Each LHU is managed and governed by a general manager appointed by the regional department of health, based on his/her professional qualifications and technical skills. This general manager appoints a financial manager and a medical director.

Services are structured according to a typical division-based model. Each division has financial autonomy over, and technical responsibility for one of three different health care system areas (Legislative decrees No. 502/1992 and No. 229/1999): directly-managed acute care and rehabilitation hospitals, health districts, and health promotion.

### 2.3. The Veneto Region’s AMI Network

The management of Acute Myocardial Infarction (AMI) has been tackled from an organizational standpoint by adopting a ‘hub and spoke’ organizational model since 2008 [23]. The approach is based on the consensus document approved by the Italian Association of Hospital Cardiologists (Associazione Nazionale Medici Cardiologi Ospedalieri, ANMCO) and the Italian Society for Telemedicine (Società Italiana per la salute digitale e la Telemedicina, SIT) [24].

A characteristic of the Veneto Region’s network lies in allowing a selective referral from satellite cardiology units (spokes) to cardiac surgical centers (hubs) with a CCL that operates 24 h a day and takes patients for primary PCI immediately.

A pre-hospital 12-lead ECG is remotely transmitted from every ambulance and every ER to the nearest cardiology unit, where the ECG-based diagnosis can be confirmed by skilled operators with direct access to the CCL at the hub hospital, possibly with fast tracks to bypass the ER, or Coronary Care Unit, or Cardiac Intensive Care Unit. A particular feature of this organizational model is that it goes beyond the borders of the single Local Health Districts: patients are admitted to the nearest CCL anywhere in the Veneto Region, considering only the geographical distance and journey time.

This model is based on the assumption that: (i) primary PCI performed as fast as possible and in good time is the preferred treatment strategy for ST-Elevation Myocardial Infarction (STEMI), as recommended by the European Society of Cardiology (ESC); (ii) the delay due to health care system organizational issues can be reduced by implementing a network enabling spoke hospitals to transfer patients promptly to a hub center for PCI, and by ensuring an efficient ambulance transfer service if the Cardiac Catheterization Laboratory (CCL) can be activated by the ambulance en route to hospital; (iii) high-level, expensive skills are needed for certain situations and complex diseases, and cannot be made available everywhere, but should be concentrated instead at highly-specialized regional centers (hubs) to which patients from local hospitals (spokes) can be promptly referred.

### 2.4. Materials

A retrospective cohort study was conducted using hospital discharge records (HDRs) from both public and private hospitals. We included residents or non-residents admitted to hospital for AMI who were discharged from any hospital operating under the National Health System (NHS) in the Veneto Region (north-east Italy) between the 1 January 2007 and the 31 December 2016.

The anonymized data obtainable from HDRs include, among others, a patient’s gender, date of birth, and citizenship, and details of the type of admission, such as discharge date, length of stay, dates of interventions, discharge status, and codes for diagnoses and procedures according to the International Classification of Diseases, Ninth Revision, Clinical Modification (ICD-9-CM), with the 24th version of the DRG Grouper. We classified citizenship as Italian, citizenship of Highly-Developed Countries (HDC), or citizenship of High Migratory Pressure Countries (HMPC). The HMPC included new Member States of the European Union, countries in Central-Eastern Africa, Asia (except for Israel, the People’s Democratic Republic of Korea, and Japan), and Central and South America; by extension, stateless individuals were also included in this group. The HDC included the other European countries, North America, Oceania, Israel and Japan.

Inclusion criteria were adopted to examine the performance of the Veneto regional network specifically in the management of STEMI. We therefore considered the ICD-9-CM diagnostic codes 410.x1 as the principal diagnosis to define AMI (initial episode of care): STEMI was identified using codes 410.01, 410.11, 410.21, 410.31, 410.41, 410.51, 410.61, 410.81 and 410.91, and non-STEMI AMI (NSTEMI) using code 410.71. Patients who underwent Coronary Artery Bypass Graft (CABG) surgery (ICD-9-CM code 36.1x) were excluded.

The study complied with the Helsinki Declaration and Italian privacy legislation (n. 196/2003). Resolution n. 85/2012 of the Guarantor for the protection of personal data has recently confirmed that anonymized personal data may be processed for medical, biomedical and epidemiological research purposes, and data concerning health status may be used in aggregate form in scientific studies.

### 2.5. Performance Indicators

Two primary indicators describing the performance of the dedicated network were considered, one for care processes and one for outcomes, using as dependent variables:

- The proportion of patients with STEMI treated with PCI within 24 h, since this was considered the treatment of choice in cases of STEMI; and
- The proportion of in-hospital deaths among patients treated for STEMI.

We used the following ICD-9-CM procedure codes to define PCI: 00.66, 36.01, 36.02, 36.05, 36.06 and 36.07. The Regional Hospital Information System reports the date, but not the exact time when procedures are performed so, for the purposes of this study, it was assumed that PCI was performed within 24 h if it was done on the day of admission to hospital.

### 2.6. Statistical Analysis

AMI hospitalization rates were calculated based on the size of the population in every year of the study period, and are reported as the rates per 100,000 population. Direct age and gender adjustments of in-hospital admissions for AMI (per 100,000 population) by type of AMI were performed using the data for the population in 2012.

Descriptive statistics were obtained for the variable investigated. Patients’ characteristics are presented as means and standard deviations (SD), for continuous variables, and using absolute and relative frequencies for categorical data.

We examined the two performance indicators by subgroup for gender (men and women), age (<45, 45–64, 65–74, 75–84, >84), and citizenship, for each year between 2007 and 2016. The annual percent changes (APC) and relative confidence intervals (CIs) were calculated to identify trends for all the variables investigated.

Two predictive models were built to estimate the relationships between the performance indicators and socio-demographic factors and, to take the hierarchical structure of the data into account, we performed multilevel logistic regression with random intercepts for hospitals, controlling for potential intra-hospital correlations. Next, to investigate disparities in outcomes by gender, age, and citizenship, we pooled the calendar years into two periods, before (2007–2008) and after (2009–2016) the introduction of the network. All statistical analyses were performed using STATA software, version 12.1 (Stata Corp LP, College Station, TX, USA). All *p*-values reported are two-sided, with a significance threshold of *p* < 0.05.

## 3. Results

Overall, there were 65,261 hospitalizations with a principal diagnosis at discharge of AMI (initial episode of care) in the decade investigated, among all hospitals operating under the NHS in the Veneto Region (north-east Italy), 55.2% of them (36,035) were STEMI, and 44.8% (29,226) were NSTEMI. The overall mean age of this patient population was 71.1 ± 13.7 years (data not shown).

**Figure 1:**
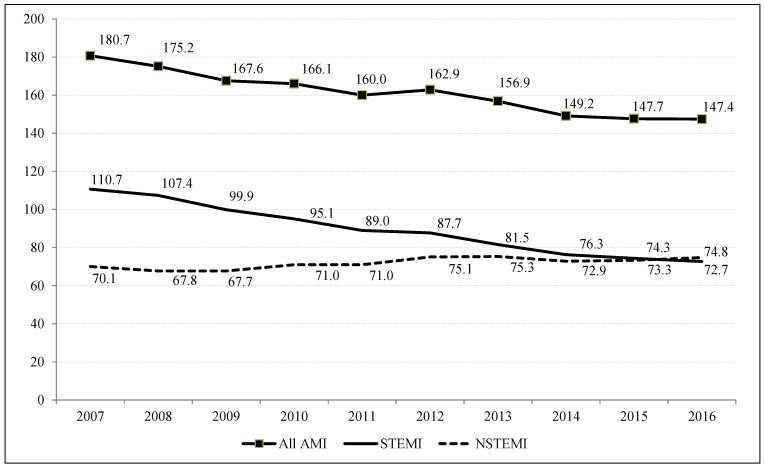
Veneto Region 2007–2016. Temporal trends in hospital admissions for AMI * (per 100,000 population), initial episode of care, by type of AMI, after direct standardization (reference population: 2012). AMI: acute myocardial infarction. * APC (CI 95%), *p*-value ➔ All STEMI = −2.3 (−2.6;−1.9), *p* < 0.001; STEMI = −4.8 (−5.3;−4.4), *p* < 0.001; NSTEMI = 1.1 (0.4;1.7), *p* < 0.001.

Figure 1 shows that the incidence of NSTEMI exceeded that of STEMI in the last year of the decade (74.8 vs. 72.7 cases per 100,000 population). This phenomenon was possibly due to a simultaneous decrease in cases of STEMI (−34%; APC:C-4.8 [−5.3;−4.4], *p* < 0.001), and increase in NSTEMI (+7%; APC: 1.1 [0.4;1.7], *p* < 0.001). On the whole, the absolute number of hospital admissions for AMI dropped by 18% (APC: −2.3 [−2.6;−1.9], *p* < 0.001) over the decade.

The mean age of the STEMI population was 70.1 ± 14.3 years, and males accounted for nearly two thirds of the patients hospitalized with STEMI (men: 66.2%; women: 33.8%). The majority of patients were of Italian citizenship (95.5%) (Table 1). In the decade from 2007 to 2016, 44.6% of patients admitted to hospital for STEMI as their principal diagnosis underwent a PCI within 24 h of admission; and the in-hospital mortality rate for STEMI was 12.2% (Table 1).

Table 2 shows the proportion of patients with STEMI treated with PCI within 24 h by socio-demographic factors and year of the study. Throughout the period investigated, this care process indicator was higher for men than for women (e.g., 38.1% vs. 18.6% in 2007), and it increased significantly over time for both genders, and especially for women, though the difference between genders was not statistically significant (APC: 6.3 [5.4;7.3] for men, and APC: 8.6 [6.2;11.1] for women). When age groups were considered, a statistically significant rising trend emerged over the ten-year period, which was statistically far more consistent for patients over 75 years old (APC, 75–84 y: 9.8; >85 y: 12.5) than for younger patients (APC, <45 y: 3.3; 45–64: 4.9). As regards citizenship, the significantly rising trend in the proportion of patients with STEMI treated with PCI within 24 h was confirmed, with no significant differences between the three citizenship groups identified (APC, Italian: 7.2; HDC: 6.1; HMPC: 5.9).

Table 3 shows the proportion of in-hospital deaths among patients treated for STEMI during the period considered, for all the subgroups investigated. Women with STEMI always had a higher in-hospital mortality rate than men (e.g., 18.9% vs. 9.9% in 2007). From 2007 to 2016, the overall observed in-hospital mortality rate declined for both genders, with no significant difference between the trends of the two groups (APC: men, −1.9 [−3.7;0.0]; women, −1.2 [−3.1;0.6]). When the different age groups were analyzed, it emerged that age had a fundamental impact on the chances of PCI within 24 h, but there was no significant difference between the declining mortality trends for the various age groups, except for the 75- to 84-year-olds (APC: −3.0 [−4.5;−1.6]). The same declining mortality trend was apparent for the citizenship subgroups too, leading to a difference that was significant for Italian patients (APC: −1.9 [−3.2;−0.6]), and greater—though it failed to reach statistical significance—for patients from HDC countries (APC: −4.9 [−12.6;3.5]).

**Figure 2:**
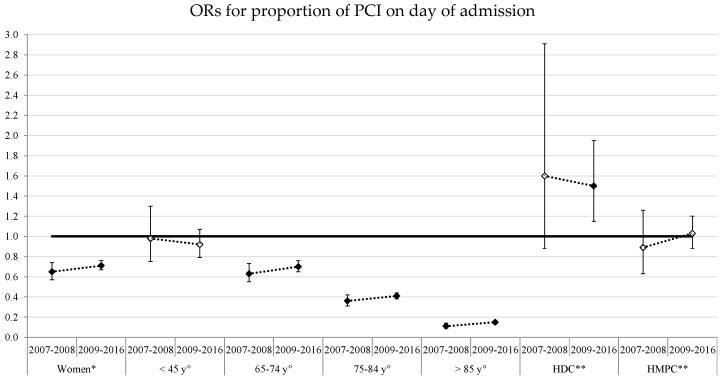

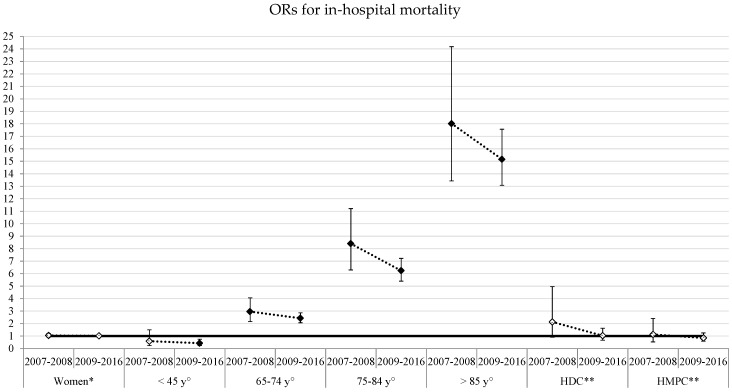
Veneto Region 2007–2016. Multilevel logistic regression analysis of associations between the two indicators investigated and socio-demographic factors; ORs and 95% confidence intervals, *p* value °°. PCI: percutaneous coronary intervention. * Reference: Men. ° Reference: 45–64 y. ** Reference: Italian. °° full indicator ➔ *p* < 0.05.

Figure 2 show the results of multilevel logistic regression analyses and the significant associations between the two performance indicators and the different socio-demographic factors, before (2007–2008) and after (2009–2016) the introduction of the network.

Although disparities persisted between genders and age groups, the multivariate analyses confirmed the trend towards a null value in almost all the different categories of socio-demographic factors (where a value of 1 attests to no differences between the factors).

## 4. Discussion

The present study showed that the proportion of PCI performed within 24 h of admission to hospital with STEMI increased considerably over the decade from 2007 to 2016 for all socio-demographic patient subgroups in the Veneto Region. A greater standardization also reduced disparities in access to PCI by gender and age group. The study also revealed no disparities in access to PCI by citizenship, neither before nor after the implementation of the regional network. During the period studied, there was a significant reduction of the related mortality among Italians, and for people aged 75–84 years.

Overall, we noted a substantial reduction in the number of cases of AMI over time, and this reduction was entirely attributable to declining numbers of STEMI, while the incidence of NSTEMI did not show any noteworthy changes, neither by year, nor over the decade as a whole. This finding is consistent with recent national [25] and international literature [26,27,28,29,30], which attributes a decrease in the incidence of STEMI to improvements in primary prevention measures and a greater use of evidence-based therapeutic changes.

Our study results demonstrate that a “hub and spoke” organizational model could increase the proportion of patients given reperfusion treatment on the day of their admission to hospital, and this applied to all the population subgroups considered. These findings are in line with reports from the United States, where hospitals implementing the key care processes (prehospital CCL activation, a single-call protocol for transfer from facilities without PCI capabilities, bypassing the ER for patients presenting directly to emergency medical services or being transferred) achieved shorter median reperfusion delays than other hospitals [31]. On the other hand, our study only found an impact on the AMI-related mortality rates in some subgroups (Italians citizens, and 75- to 84-year-olds). Though information on the exact door-to-balloon (DTB) time was unavailable for our sample, a possible interpretation for this limited impact may be that the speed of response is actually still outside the critical time-window, and having increased the proportion of PCI performed within 24 h may have no major impact on survival [32,33]. Our data are partly consistent with the findings of a previous study [34], in which the authors documented a marked reduction in median DTB time and a greater compliance with the guidelines, but found in-hospital mortality unchanged. In other words, the successful implementation of organizational efforts to optimize the quality of this care process did not produce the expected survival benefit. Another recent study by Wang et al. on 5881 patients with STEMI undergoing PCI showed that the median DTB time was 101 min from January to December 2005, and dropped to 87 min from July 2006 to June 2007, but this shorter DTB time was not associated with any improvement in mortality [35].

The other aim of our study was to see whether the new network reduces health inequities among socio-demographic subgroups of the population, with a view to ensuring that the structural and procedural features of health service delivery result in an equitable distribution of the services for individuals and population subgroups with comparable needs and wants [36].

Regarding citizenship, we found a statistically significant increase from 2007 to 2016 in the number of PCI performed within 24 h in all categories of citizens, and there were no disparities between them even before the network was implemented. Similarly, a study conducted in the Netherlands [37] found no ethnic inequities in the revascularization rates after STEMI, and a UK study found none in the revascularization rates after AMI [38]. These findings contrast with the abundance of literature from the USA showing considerably lower revascularization rates after coronary events for African Americans compared with White Americans [39,40,41]. A major difference between Italy, the UK or the Netherlands and the USA lies in the former countries’ universal health care provision instead of private health insurance schemes that fail to cover the whole population’s health care needs. In fact, Italy and the UK have health systems funded by taxes that guarantee a standard level of care for everyone, and The Netherlands obliges all residents to have health insurance by law. Our study also found citizens from HDC more likely to receive prompt treatment than Italians after the network was reorganized, and this could be because the country of origin’s reimbursement of the costs for these patients’ treatment is allied to the demonstration of a strict adherence to the guidelines.

After the implementation of the network, there was a statistically significant increase (from 2007 to 2016) in the proportion of PCI performed within 24 h. This was true of both genders, with a greater improvement in women than in men (APC 8.6 vs. 6.3), though the difference between genders was not statistically significant. Multivariate analysis nonetheless showed that gender disparities in STEMI management persisted after the network’s implementation. This is consistent with the gender disparities seen in our previous studies [42,43,44]. This inequity in the administration of the evidence-based best treatment for STEMI might be partly attributable to a greater delay before women access hospital care, which would prevent the adoption of a PCI approach. Whatever its cause, such disparities are a health system concern that can be solved by associating suitable strategies with the network model. In fact, even if high-quality care were accessible to everyone, there is no guaranteeing that all patients would benefit equally, so strategies are needed to ensure that patients are all aware of how to make best use of the health care services available.

We also found that the network significantly improved access to PCI within 24 h for all age groups, and especially for the previously disadvantaged older age groups (>65 years old). We only noted a significant reduction in mortality for the 75- to 84-year-olds, however, during the period examined. Despite these improvements, multivariate analysis showed that some degree of age-related inequity persisted. These results are in line with a similar study conducted in Switzerland [45], where elderly patients (and females) were at a disadvantage in the circadian provision of primary PCI in a cohort of patients with AMI. The Swiss authors surmised that physicians and other hospital staff may slow the process down for some patients more than others and, somewhere along the line, staff members make decisions about patients based on patient group membership rather than guidelines. There is no reason to believe that this reflects a pattern of deliberate behavior, however, since many studies document the presence of unconscious bias among medical personnel, and the effects these have on their decisions and actions [46,47].

More generally speaking, Peterson et al. [48] also analyzed data on approximately 2.5 million patients with STEMI admitted to US hospitals between 1990 and 2006, finding that—despite very clear, and widely-adopted ACCC/AHA STEMI and NSTEMI guidelines—there were still disparities in STEMI treatment for women, blacks, and elderly patients. The results of studies conducted specifically to test adherence to, and effectiveness of standardized guidelines show that, when they are in place and enforced, standardized protocols reduce disparities and improve patient outcomes [49]. The present study underscores the need to monitor the implementation of organizational models and/or standardized protocols by means of up-to-date administrative datasets to see whether a given strategy reduces health care disparities. In fact, hospital administration databases are of considerable practical value for the purpose of broad-based quality assessments, and could be useful for monitoring health equity issues too, identifying problems and targeting areas that might require more in-depth investigations based on more specific data.

The main strength of our study lies in that it was population-based, minimizing selection bias and relying on independently-collected data. Our study also has some limitations, however. First, it was an observational study, which limits our ability to draw causal inferences. In particular, we cannot disentangle whether the disparities arose: because the quality of care provided by the health care services differed according to patients’ sociodemographic characteristics; or because patients’ sociodemographic factors influenced their attitudes or behavior, or the clinical characteristics predisposing them to different treatment for acute events. That said, studies on disparities can only be observational.

A second limitation lies in that, despite their wide use as a valuable source for health care research on specific disease, hospital discharge data have some drawbacks in terms of their diagnostic coding accuracy (in our case, the use of the ICD-9-CM codes to identify the AMI). These codes have the advantage of being widely available and being less effortful and costly than consulting medical charts, but their effective use hinges on how reliably the codes reflect a given patient’s clinical condition.

Finally, cardiovascular risk is related to many economic, social and predisposing factors, apart from age and gender. Unfortunately, our health administration records do not track all the important risk factors for AMI, such as lifestyle indicators, which could have an important influence on survival. That said, such risk factors could only have a confounding effect if they are differently associated with both outcome and exposure.

## 5. Conclusions

In conclusion, our study confirms the effectiveness of the new hub-and-spoke organizational model of the Veneto Region’s network for AMI in facilitating primary PCI on STEMI and reducing in-hospital mortality. The present study also emphasizes that the implementation of organizational models and/or standardized protocols should be monitored with the aid of up-to-date administrative datasets to see whether a given strategy reduces health care disparities.

Based on such indicators, the present study revealed numerous opportunities for improvements and adaptations to the intervention, and for regularly collecting process measures. At the same time, it will be helpful not only to use administrative databases, but also to introduce qualitative assessment methods (e.g., surveys, interviews) to identify problems and target areas that might require more in-depth investigations based on more specific data.

Even though the new network improved the process of care for STEMI and reduced disparities in this process between all population subgroups, this did not generate the expected survival benefit in all patient subgroups.

The present findings could prompt policy-makers to seriously consider the feasibility of designing and implementing programs for health care providers, organizations, and community groups, and policies that focus not only on patients. Finally, sharing feedback with providers, and incentivizing them to reduce disparities could have an important role.

## Figures and Tables

**Table 1:** Veneto Region 2007–2016. Characteristics of hospital admissions for STEMI, in-hospital mortality and proportion of PCI on day of admission by gender, age, and citizenship.

	Hospital Admissions		Mean Age	Average Hospital Stay	PCI on Day of Admission		In-Hospital Mortality	
	*n*	%	(y) ± SD	d ± SD	*n*	%	*n*	%
**Total**	36,035	-	70.1 ± 14.3	9.24 ± 8.80	16,075	44.6	4385	12.2
**Gender**								
**Male**	23,862	66.2	66.2 ± 13.5	8.82 ± 8.75	12,284	51.5	2162	9.1
**Female**	12,173	33.8	77.7 ± 12.7	10.04 ± 8.83	3791	31.1	2223	18.3
**Age**								
**<45 y**	1424	3.9	40.0 ± 4.4	7.34 ± 8.44	844	59.3	18	1.3
**45–64 y**	11,415	31.7	55.9 ± 5.4	7.61 ± 6.51	7012	61.4	312	2.7
**65–74 y**	7949	22.1	69.6 ± 2.9	9.19 ± 8.96	4039	50.8	536	6.7
**75–84 y**	8771	24.3	79.6 ± 2.9	10.90 ± 10.63	3147	35.9	1432	16.3
**>84 y**	6476	18.0	89.4 ± 3.7	10.33 ± 8.86	1033	16.0	2087	32.2
**Citizenship**								
**Italian**	34,414	95.5	70.7 ± 14.0	8.4 ± 7.3	15,130	44.0	4314	12.5
**HDC**	394	10.9	62.9 ± 11.8	8.2 ± 10.4	262	66.5	29	7.4
**HMPC**	1127	3.1	52.7 ± 12.2	11.8 ± 10.9	645	57.2	38	3.4

PCI: percutaneous coronary intervention; HDC: highly-developed countries; HMPC: high migratory pressure countries.

**Table 2:** Veneto Region 2007–2016. Proportion of PCI within 24 h of hospital admission for STEMI by gender, age, and citizenship.

	% of PCI Within 24 h										
	2007 (*n* = 4040)	2008 (*n* = 4083)	2009 (*n* = 3908)	2010 (*n* = 3793)	2011 (*n* = 3644)	2012 (*n* = 3536)	2013 (*n* = 3362)	2014 (*n* = 3252)	2015 (*n* = 3228)	2016 (*n* = 3189)	APC (CI 95%)
**Gender**											
Male	38.1	40.8	42.5	48.3	49.9	54.4	59.0	61.0	62.2	64.2	6.3 (5.4;7.3) °
Female	18.6	22.5	28.6	28.5	32.4	32.9	38.2	38.8	40.8	40.6	8.6 (6.2;11.1) °
**Age**											
<45 y	51.3	47.0	60.9	53.0	58.8	63.4	69.4	69.7	66.7	59.6	3.3 (0.9;5.8) °
45–64 y	47.8	49.9	53.2	61.2	60.9	63.2	68.7	68.9	71.8	71.9	4.9 (3.8;6.0) °
65–74 y	35.2	39.2	43.6	44.5	50.6	54.8	59.2	61.4	63.4	64.5	7.2 (6.0;8.4) °
75–84 y	22.0	25.9	27.5	32.8	35.9	37.7	44.9	47.5	47.8	50.5	9.8 (8.2;11.5) °
>84 y	8.0	9.4	12.6	14.9	14.9	19.3	17.3	21.9	22.1	24.2	12.5 (9.4;15.6) °
**Citizenship**											
Italian	30.4	33.8	37.2	40.8	43.7	46.9	51.1	53.1	54.7	56.2	7.2 (6.1;8.3) °
HDC	50.0	46.4	68.3	46.0	62.9	61.3	92.5	77.3	74.5	76.3	6.1 (1.8;10.6) °
HMPC	41.1	44.6	45.5	60.0	54.3	52.3	65.3	66.1	63.4	70.9	5.9 (3.8;8.1) °

PCI: percutaneous coronary intervention; APC: annual percent change; HDC: highly-developed countries; HMPC: high migratory pressure countries. ° *p* < 0.05.

**Table 3:** Veneto Region 2007–2016. In-hospital mortality after hospital admission for STEMI by gender, age, and citizenship.

	% in-Hospital Mortality										
	2007 (*n* = 4040)	2008 (*n* = 4083)	2009 (*n* = 3908)	2010 (*n* = 3793)	2011 (*n* = 3644)	2012 (*n* = 3536)	2013 (*n* = 3362)	2014 (*n* = 3252)	2015 (*n* = 3228)	2016 (*n* = 3189)	APC (CI 95%)
**Gender**											
Male	9.9	8.7	9.8	9.0	9.6	9.3	8.5	9.8	8.2	7.5	−1.9 (−3.7;0.0)
Female	18.9	19.4	16.7	18.4	20.6	17.8	19.4	16.6	17.7	16.2	−1.2 (−3.1;0.6)
**Age**											
<45 y	1.3	1.8	-	0.7	3.3	0.6	0.8	1.6	1.8	1.0	−1.6 (−16.0;15.3) *
45–64 y	3.1	1.9	2.6	2.5	2.7	2.7	3.0	4.1	2.3	2.5	1.4 (−3.9;6.9)
65–74 y	7.7	6.5	6.4	7.2	8.1	7.3	6.0	7.2	5.9	4.8	−3.0 (−6.2;0.3)
75–84 y	18.1	17.6	17.3	16.1	16.8	17.9	15.3	14.1	15.1	13.1	−3.0 (−4.5;−1.6) °
>84 y	31.2	34.0	31.7	31.1	35.3	29.5	35.3	31.6	31.6	31.1	−0.2 (−1.8;1.4)
**Citizenship**											
Italian	13.5	12.9	12.4	12.7	13.6	12.6	12.6	12.4	11.6	10.4	−1.9 (−3.2;−0.6) °
HDC	15.6	7.1	7.3	4.0	8.6	6.5	7.5	6.8	7.3	5.3	−4.9 (−12.6;3.5)
HMPC	5.3	3.0	4.0	3.6	1.7	3.6	1.6	3.3	3.3	4.7	−1.8 (−11.5;8.9)

APC: annual percent change; HDC: highly-developed countries; HMPC: high migratory pressure countries. ° *p* < 0.05. * excluded year 2009.

## References

[1] WHO Regional Office for Europe (2006). Gaining Health: The European Strategy for the Prevention and Control of Noncommunicable Diseases.

[2] Townsend N., Wilson L., Bhatnagar P., Wickramasinghe K., Rayner M., Nichols M. (2016). Cardiovascular disease in Europe: Epidemiological update 2016. Eur. Heart J..

[3] Hartley A., Marshall D.C., Salciccioli J.D., Sikkel M.B., Maruthappu M., Shalhoub J. (2016). Trends in mortality from ischemic heart disease and cerebrovascular disease in Europe: 1980 to 2009. Circulation.

[4] Amit G., Cafri C., Gilutz H., Ilia R., Zahger D. (2007). Benefit of direct ambulance to coronary care unit admission of acute myocardial infarction patients undergoing primary percutaneous intervention. Int. J. Cardiol..

[5] Andersen H.R., Nielsen T.T., Rasmussen K., Thuesen L., Kelbaek H., Thayssen P., Abildgaard U., Pedersen F., Madsen J.K., Grande P. (2003). DANAMI-2 Investigators. A comparison of coronary angioplasty with fibrinolytic therapy in acute myocardial infarction. N. Engl. J. Med..

[6] Terkelsen C.J., Lassen J.F., Nørgaard B.L., Gerdes J.C., Poulsen S.H., Bendix K., Ankersen J.P., Gøtzsche L.B., Rømer F.K., Nielsen T.T. (2005). Reduction of treatment delay in patients with ST-elevation myocardial infarction: Impact of pre-hospital diagnosis and direct referral to primary percutanous coronary intervention. Eur. Heart J..

[7] Dorsch M.F., Greenwood J.P., Priestley C., Somers K., Hague C., Blaxill J.M., Wheatcroft S.B., Mackintosh A.F., McLenachan J.M., Blackman D.J. (2008). Direct ambulance admission to the cardiac catheterization laboratory significantly reduces door-to-balloon times in primary percutaneous coronary intervention. Am. Heart J..

[8] Brown J.P., Mahmud E., Dunford J.V., Ben-Yehuda O. (2008). Effect of prehospital 12-lead electrocardiogram on activation of the cardiac catheterization laboratory and door-to-balloon time in ST-segment elevation acute myocardial infarction. Am. J. Cardiol..

[9] Quinn T., Johnsen S., Gale C.P., Snooks H., McLean S., Woollard M., Weston C., Myocardial Ischaemia National Audit Project (MINAP) Steering Group (2014). Effects of prehospital 12-lead ECG on processes of care and mortality in acute coronary syndrome: A linked cohort study from the Myocardial Ischaemia National Audit Project. Heart.

[10] Edmund Anstey D., Li S., Thomas L., Wang T.Y., Wiviott S.D. (2016). Race and sex differences in management and outcomes of patients after ST-elevation and non-ST-elevation myocardial infarct: Results From the NCDR. Clin. Cardiol..

[11] Canto J.G., Allison J.J., Kiefe C.I., Fincher C., Farmer R., Sekar P., Person S., Weissman N.W. (2000). Relation of race and sex to the use of reperfusion therapy in Medicare beneficiaries with acute myocardial infarction. N. Engl. J. Med..

[12] Vaccarino V., Rathore S.S., Wenger N.K., Frederick P.D., Abramson J.L., Barron H.V., Manhapra A., Mallik S., Krumholz H.M., National Registry of Myocardial Infarction Investigators (2005). Sex and racial differences in the management of acute myocardial infarction, 1994 through 2002. N. Engl. J. Med..

[13] Hagen T.P., Häkkinen U., Iversen T., Klitkou S.T., Moger T.A., EuroHOPE Study Group (2015). Socio-economic inequality in the use of procedures and mortality among AMI patients: Quantifying the effects along different paths. Health Econ..

[14] Mensah G.A. (2005). Eliminating disparities in cardiovascular health: Six strategic imperatives and a framework for action. Circulation.

[15] Colais P., Agabiti N., Fusco D., Pinnarelli L., Sorge C., Perucci C.A., Davoli M. (2013). Inequality in 30-day mortality and the wait for surgery after hip fracture: The impact of the regional health care evaluation program in Lazio (Italy). Int. J. Qual. Health Care.

[16] Huded C.P., Johnson M., Kravitz K., Menon V., Abdallah M., Gullett T.C., Hantz S., Ellis S.G., Podolsky S.R., Meldon S.W. (2018). 4-Step Protocol for Disparities in STEMI Care and Outcomes in Women. J. Am. Coll. Cardiol..

[17] Glickman S.W., Granger C.B., Ou F.S., O’Brien S., Lytle B.L., Cairns C.B., Mears G., Hoekstra J.W., Garvey J.L., Peterson E.D. (2010). Impact of a statewide ST-segment-elevation myocardial infarction regionalization program on treatment times for women, minorities, and the elderly. Circ. Cardiovasc. Qual. Outcomes.

[18] Evans L.W., van Woerden H., Davies G.R., Fone D. (2016). Impact of service redesign on the socioeconomic inequity in revascularisation rates for patients with acute myocardial infarction: A natural experiment and electronic record-linked cohort study. BMJ Open.

[19] (2018). Demografia in Cifre [Demography in Numbers][Website]; Rome: Istituto Nazionale di Statistica. http://demo.istat.it/.

[20] Istituto Nazionale di Statistica [National Institute of Statistics][Website] Rome: National Institute of Statistics. 2017. http://dati.istat.it.

[21] AUR—Anagrafe Unica Assistiti Regionale (2011). *Portale Sanità Regione del Veneto* [*Website*]; Venice: Veneto Region, Italy. https://salute.regione.veneto.it/web/bando-ssi/aur-anagrafe-unica-assistiti-regionale.

[22] World Health Organization The Veneto Model—A Regional Approach to Tackling Global and European Health Challenges (2016). http://www.euro.who.int/en/publications/abstracts/the-veneto-model-a-regional-approach-to-tackling-global-and-european-health-challenges-2016.

[23] Regione del Veneto; Bur n. 15 del 19 Febbraio 2008; Materia: Sanità e Igiene Pubblica; Deliberazione della Giunta Regionale n. 4550 del 28 Dicembre 2007 n.d. https://bur.regione.veneto.it/BurvServices/pubblica/DettaglioDgr.aspx?id=203192.

[24] Documento di, Consenso (2005). La rete interospedaliera per l’emergenza coronarica. Ital. Health J..

[25] Barchielli A., Profili F., Balzi D., Francesconi P., Zuppiroli A., Cipriani F. (2015). Trends in occurrence, treatment, and outcomes of acute myocardial infarction in Tuscany Region (Central Italy), 1997–2010. Epidemiol. Prev..

[26] Sugiyama T., Hasegawa K., Kobayashi Y., Takahashi O., Fukui T., Tsugawa Y. (2015). Differential time trends of outcomes and costs of care for acute myocardial infarction hospitalizations by ST elevation and type of intervention in the United States, 2001–2011. J. Am. Heart Assoc..

[27] McManus D.D., Gore J., Yarzebski J., Spencer F., Lessard D., Goldberg R.J. (2011). Recent trends in the incidence, treatment, and outcomes of patients with STEMI and NSTEMI. Am. J. Med..

[28] Yeh R.W., Sidney S., Chandra M., Sorel M., Selby J.V., Go A.S. (2010). Population trends in the incidence and outcomes of acute myocardial infarction. N. Engl. J. Med..

[29] Chen J., Normand S.L., Wang Y., Drye E.E., Schreiner G.C., Krumholz H.M. (2010). Recent declines in hospitalizations for acute myocardial infarction for Medicare fee-for-service beneficiaries: Progress and continuing challenges. Circulation.

[30] Luepker R.V., Berger A.K. (2010). Is acute myocardial infarction disappearing?. Circulation.

[31] Fordyce C.B., Al-Khalidi H.R., Jollis J.G., Roettig M.L., Gu J., Bagai A., Berger P.B., Corbett C.C., Dauerman H.L., Fox K. (2017). STEMI Systems Accelerator Project. Association of rapid care process implementation on reperfusion times across multiple ST-segment-elevation myocardial infarction networks. Circ. Cardiovasc. Interv..

[32] Gersh B.J., Stone G.W., White H.D., Holmes D.R. (2005). Pharmacological facilitation of primary percutaneous coronary intervention for acute myocardial infarction: Is the slope of the curve the shape of the future?. JAMA.

[33] Reimer K.A., Lowe J.E., Rasmussen M.M., Jennings R.B. (1977). The wavefront phenomenon of ischemic cell death. 1. Myocardial infarct size vs duration of coronary occlusion in dogs. Circulation.

[34] Flynn A., Moscucci M., Share D., Smith D., LaLonde T., Changezi H., Riba A., Gurm H.S. (2010). Trends in door-to-balloon time and mortality in patients with ST-elevation myocardial infarction undergoing primary percutaneous coronary intervention. Arch. Intern. Med..

[35] Wang T.Y., Fonarow G.C., Hernandez A.F., Liang L., Ellrodt G., Nallamothu B.K., Shah B.R., Cannon C.P., Peterson E.D. (2009). The dissociation between door-to-balloon time improvement and improvements in other acute myocardial infarction care processes and patient outcomes. Arch. Intern. Med..

[36] Aday L.A., Begley C.E., Lairson D.R., Slater C.H., Richard A.J., Montoya I.D. (1999). A framework for assessing the effectiveness, efficiency, and equity of behavioral healthcare. Am. J. Manag. Care.

[37] Van Oeffelen A.A., Rittersma S., Vaartjes I., Stronks K., Bots M.L., Agyemang C. (2015). Are there ethnic inequalities in revascularisation procedure rate after an ST-elevation myocardial infarction?. PLoS ONE.

[38] Bansal N., Fischbacher C.M., Bhopal R.S., Brown H., Steiner M.F., Capewell S., Scottish Health and Ethnicity Linkage Study (2013). Myocardial infarction incidence and survival by ethnic group: Scottish Health and Ethnicity Linkage retrospective cohort study. BMJ Open.

[39] Freund K.M., Jacobs A.K., Pechacek J.A., White H.F., Ash A.S. (2012). Disparities by race, ethnicity, and sex in treating acute coronary syndromes. J. Womens Health.

[40] Iribarren C., Tolstykh I., Somkin C.P., Ackerson L.M., Brown T.T., Scheffler R., Syme L., Kawachi I. (2005). Sex and racial/ethnic disparities in outcomes after acute myocardial infarction: A cohort study among members of a large integrated health care delivery system in northern California. Arch. Intern. Med..

[41] Thomas K.L., Honeycutt E., Shaw L.K., Peterson E.D. (2010). Racial differences in long-term survival among patients with coronary artery disease. Am. Heart J..

[42] Buja A., Canavese D., Furlan P., Lago L., Saia M., Baldo V. (2015). Are hospital process quality indicators influenced by socio-demographic health determinants. Eur. J. Public Health.

[43] Srichaiveth B., Ruengsakulrach P., Visudharom K., Sanguanwong S., Tangsubutr W., Insamian P. (2007). Impact of gender on treatment and clinical outcomes in acute ST elevation myocardial infarction patients in Thailand. J. Med. Assoc. Thai..

[44] Falcone M., Del Santo S., Forni S., Pepe P., Marchi M., Rossi G. (2013). Equity of access to percutaneous transluminal coronary angioplasty (PTCA) among patients with acute myocardial infarction in Tuscany Region (Central Italy), 2001–2008. Epidemiol. Prev..

[45] Pilgrim T., Heg D., Tal K., Erne P., Radovanovic D., Windecker S., Jüni P., AMIS Plus Investigators (2015). Age- and gender-related disparities in primary percutaneous coronary interventions for acute ST-segment elevation myocardial infarction. PLoS ONE.

[46] Blair I.V., Steiner J.F., Havranek E.P. (2011). Unconscious (implicit) bias and health disparities: Where do we go from here?. Perm. J..

[47] Schulman K.A., Berlin J.A., Harless W., Kerner J.F., Sistrunk S., Gersh B.J., Dubé R., Taleghani C.K., Burke J.E., Williams S. (1999). The effect of race and sex on physicians’ recommendations for cardiac catheterization. N. Engl. J. Med..

[48] Peterson E.D., Shah B.R., Parsons L., Pollack CV., French W.J., Canto J.G., Gibson C.M., Rogers W.J. (2008). Trends in quality of care for patients with acute myocardial infarction in the National Registry of Myocardial Infarction from 1990 to 2006. Am. Heart J..

[49] Wang T.Y., Dai D., Hernandez A.F., Bhatt D.L., Heidenreich P.A., Fonarow G.C., Peterson E.D. (2011). The importance of consistent, high-quality acute myocardial infarction and heart failure care results from the American Heart Association’s Get with the Guidelines Program. J. Am. Coll. Cardiol..

